# Severe CTE and TDP-43 pathology in a former professional soccer player with dementia: a clinicopathological case report and review of the literature

**DOI:** 10.1186/s40478-023-01572-3

**Published:** 2023-05-10

**Authors:** Suzan van Amerongen, Suzie Kamps, Kyra K. M. Kaijser, Yolande A. L. Pijnenburg, Philip Scheltens, Charlotte E. Teunissen, Frederik Barkhof, Rik Ossenkoppele, Annemieke J. M. Rozemuller, Robert A. Stern, Jeroen J. M. Hoozemans, Everard G. B. Vijverberg

**Affiliations:** 1 Amsterdam Neuroscience, Neurodegeneration, Amsterdam, the Netherlands; 2 Department of Neurology, Amsterdam UMC, location Vrije Universiteit Amsterdam, Alzheimer Center Amsterdam, De Boelelaan 1117, 1081 HV Amsterdam, the Netherlands; 3 Department of Neurology, Boston University Alzheimer’s Disease Research Center, Boston University CTE Center, Boston University Chobanian and Avedisian School of Medicine, Boston, MA USA; 4 EQT Life Sciences, Amsterdam, the Netherlands; 5 Department of Radiology & Nuclear Medicine, Amsterdam UMC, Location Vrije Universiteit Amsterdam, Amsterdam, the Netherlands; 6 Clinical Memory Research Unit, Lund University, Lund, Sweden; 7 Queen Square Institute of Neurology and Centre for Medical Image Computing, University College London, London, UK; 8 Neurochemistry Laboratory, Department of Clinical Chemistry, Amsterdam UMC, Location Vrije Universiteit Amsterdam, Amsterdam, the Netherlands; 9 Department of Pathology, Amsterdam UMC, location Vrije Universiteit Amsterdam, Amsterdam, the Netherlands; 10 Departments of Neurosurgery, and Anatomy and Neurobiology, Boston University Chobanian and Avedisian School of Medicine, Boston, MA USA

**Keywords:** Soccer, Association football, Repetitive head impacts, Chronic traumatic encephalopathy, Tauopathy, Neurodegeneration, Biomarkers, Traumatic encephalopathy syndrome

## Abstract

In the last decades, numerous post-mortem case series have documented chronic traumatic encephalopathy (CTE) in former contact-sport athletes, though reports of CTE pathology in former soccer players are scarce. This study presents a clinicopathological case of a former professional soccer player with young-onset dementia. The patient experienced early onset progressive cognitive decline and developed dementia in his mid-50 s, after playing soccer for 12 years at a professional level. While the clinical picture mimicked Alzheimer’s disease, amyloid PET imaging did not provide evidence of elevated beta-amyloid plaque density. After he died in his mid-60 s, brain autopsy showed severe phosphorylated tau (p-tau) abnormalities fulfilling the neuropathological criteria for high-stage CTE, as well as astrocytic and oligodendroglial tau pathology in terms of tufted astrocytes, thorn-shaped astrocytes, and coiled bodies. Additionally, there were TAR DNA-binding protein 43 (TDP-43) positive cytoplasmic inclusions in the frontal lobe and hippocampus, and Amyloid Precursor Protein (APP) positivity in the axons of the white matter. A systematic review of the literature revealed only 13 other soccer players with postmortem diagnosis of CTE. Our report illustrates the complex clinicopathological correlation of CTE and the need for disease-specific biomarkers.

**Supplementary Information:**

The online version contains supplementary material available at 10.1186/s40478-023-01572-3.

## Introduction

With more than 250 million professional and recreational participants, soccer is the most popular sport worldwide. [1] As soccer involves multiple sources of head impact, such as collisions and heading, there are growing concerns regarding the long-term brain health of soccer players. In 2019, Mackay et al. reported a 3.5 times higher mortality risk of neurodegenerative diseases among former professional Scottish soccer players. [2] These results were later corroborated by findings from Russell et al. (2021), that further connected the risk for neurodegenerative diseases to outfield player position and career duration. [3] This relationship might be linked to soccer-related symptomatic concussive, and the more common asymptomatic ‘subconcussive’ head impacts and these results were consistent with mortality studies among athletes performing other contact sports. [4–7]

The link between repetitive head impacts (RHI) in sports and late-life progressive cognitive, neuropsychiatric, and motor impairments has been recognized for decades. [8] Post-mortem neuropathological evaluation has revealed unique neurodegenerative changes, known as chronic traumatic encephalopathy (CTE), in hundreds of former contact sport athletes. The unique pathological features of CTE are depositions of phosphorylated tau (p-tau) deep in the cortical sulci around small blood vessels and have been detected most frequently in the brains of American football players and boxers. [9–13] CTE in former soccer players, however, has been reported to a lesser extent and, as such, the burden of CTE in this population remains unknown. [14, 15] Given the worldwide popularity of soccer, and the fact that head impacts in soccer are different and unique compared to other contact or collision sports, it is important to investigate the occurrence of CTE and associated pathology in the brains of former soccer players.

Well-documented clinical and neuropathological information about soccer players with CTE is scarce yet important for a better understanding of this neurodegenerative disease. In the current study, we present a case report of a former professional Dutch soccer player with young-onset dementia and pathology-confirmed CTE, supported by comprehensive longitudinal clinical, neuroimaging, and fluid biomarker data. We also provide a literature overview of current evidence of CTE among soccer players.

## Case presentation

### Clinical description

**Fig. 1:**
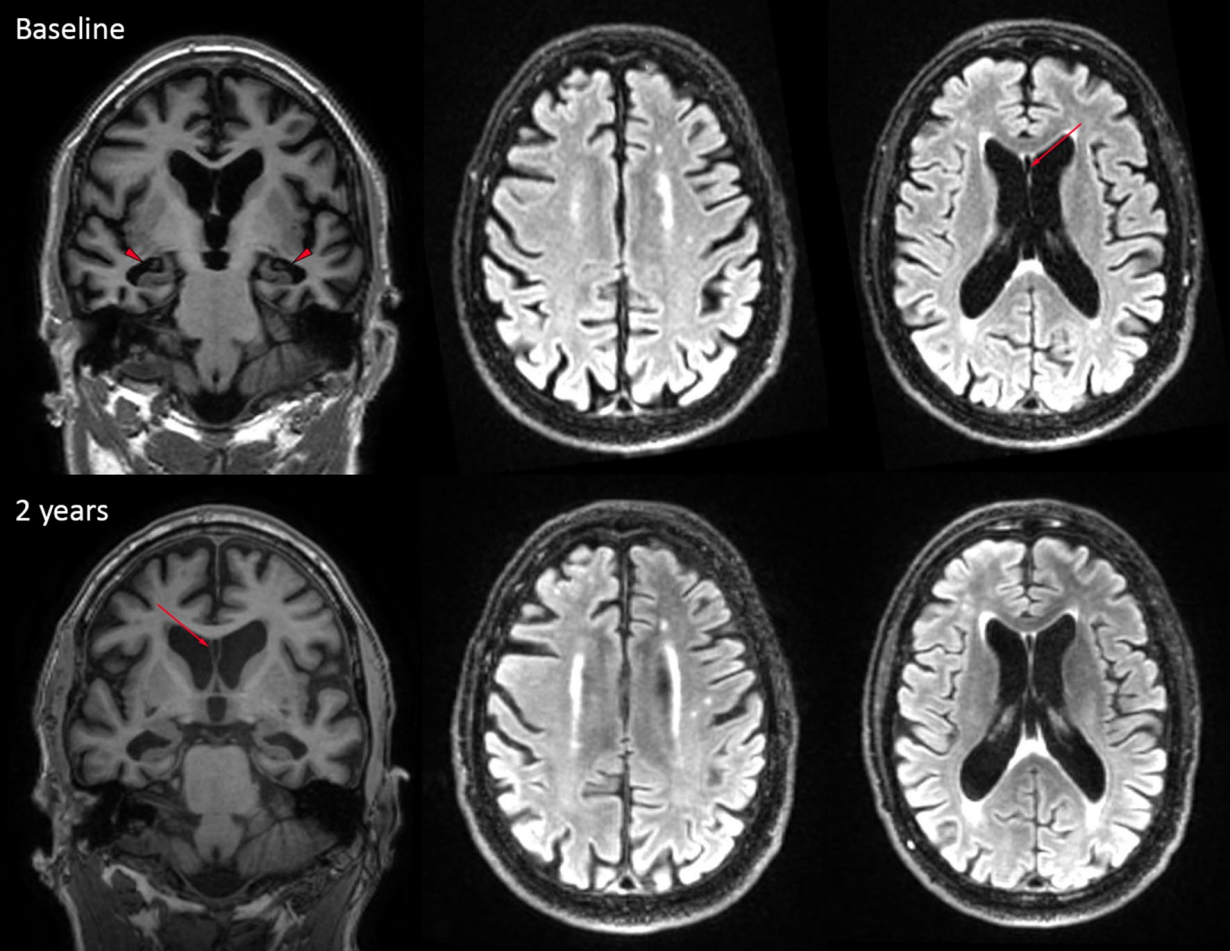
Brain MRI. Images were acquired at baseline and after two years. The coronal T1 images on the left show global atrophy (global cortical atrophy score 1–2), and severe bilateral hippocampal atrophy (medial temporal atrophy score 3, red triangle). The axial T2 FLAIR images in the middle and on the right display the white matter hyperintensities. The red arrows indicate the cavum septum pellucidum (CSP)

This male patient played soccer for 24 years, including 12 years at a professional level in the top league of the Netherlands (the 1980s), and retired at the age of 32. As a forward, he was a skilled header, and he was praised for his header goals. He also mentioned having experienced multiple collisions that involved head impact playing soccer, at least once leading to loss of consciousness. He was referred to the memory clinic of the Alzheimer Center Amsterdam at the age of 54. The referral center considered that the patient suffered from young-onset Alzheimer’s disease (AD). According to his family, the problems started around the age of 50 with progressive short-term memory complaints, leading to forgetfulness, and difficulties in household activities and financial administration. The patient was also increasingly disorientated to place. Apathy was reported, but there were no other psychiatric features, such as explosivity, impulsive behavior, emotional lability, or mood symptoms. Subsequent to his soccer career, there was no known history of traumatic brain injury. The patient had no history of any medical disease, drug abuse, or alcoholism and did not use any medication. He smoked more than 30 pack years. He reported that his father experienced mild, non-progressive memory complaints at the age of 60, but he did not have functional limitations and he never received a formal diagnosis. Further family history was negative for neurological disorders. Physical examination revealed no abnormalities. Neuropsychological assessment at baseline showed severe memory deficits, decreased processing speed, and deficits in executive functioning. The Mini-Mental State Examination (MMSE) was 23 out of 30 and the Clinical Dementia Rating (CDR) was 1, indicative of mild dementia. Neuropsychiatric symptoms were assessed via the Neuropsychiatry Inventory Questionnaire (NPI-Q) [16], revealing the presence of apathy and loss of appetite (NPI-Q total score: 12). A T1 brain MRI scan showed moderate to severe atrophy, with a bilateral medial temporal lobe atrophy score of 3, a global cortical atrophy score of 1–2 and a bilateral parietal atrophy score of 2 [17–19]. The fluid-attenuated inversion recovery (FLAIR) showed some punctate foci of white matter hyperintensities (WMH) consistent with Fazekas grade 1. [20] In addition, a cavum septum pellucidum (CSP) was visible (Fig. 1). Based on the clinical presentation, neuropsychological examination, and MRI scan, the patient was diagnosed with probable AD dementia, according to the 2011 National Institute on Aging—Alzheimer’s Association diagnostic criteria. [21] To support this diagnosis, a lumbar puncture was performed to measure AD-specific biomarkers in cerebrospinal fluid (CSF). CSF revealed normal levels of Aβ 1–42, total tau and p-tau-181; Aβ 1–42: 979 pg/ml (ref > 550 pg/ml), total tau: 324 pg/ml (ref < 375 pg/ml), p-tau-181: 40 pg/ml (ref < 52 pg/ml), measured with Innotest© assays. [22] Additionally, a negative 18F-Flutametamol positron emission tomography (PET) did not support a clinical diagnosis of Alzheimer’s disease. The patient’s APOE genotype was APOE E2/E3 and the microtubule-associated protein tau (MAPT) haplotype was H1/H1. His DNA was sequenced for the most common autosomal dominant causes of dementia, including pathogenic MAPT gene mutations and repeat expansions of chromosome 9 open reading frame 72 (C9orf72); the results were negative*.* Because of the unlikelihood of Alzheimer’s pathology as the underlying cause, and because no other disorders or conditions could fully account for the patient’s clinical presentation, a diagnosis of dementia due to CTE was suggested and the patient was followed clinically. In retrospect, using the 2021 National Institute of Neurological Disorders and Stroke (NINDS) Consensus Diagnostic Criteria for Traumatic Encephalopathy Syndrome (TES; meant to represent the clinical manifestation of CTE pathology, to be used in research settings), the patient would meet the criteria for TES with probable CTE level of certainty, because of the extensive exposure to RHI, the progressive cognitive impairment (stage mild dementia), the delayed onset, and the presence of a moderate level of apathy. [23]

After his initial visit, there was a slow deterioration over the years. A follow-up MRI scan after two years showed similar atrophy scores but increased ventricular dilatation (Fig. 1). Follow-up neuropsychological examination after two, three, and four years since the diagnosis of dementia demonstrated a further decline in memory, executive functions, and visuospatial abilities, whereas his processing speed and attention remained relatively stable. The MMSE decreased by 5 points over a four-year period (latest score: 17/30). According to the NPI-Q, more neuropsychiatric symptoms were reported during the course of the disease. The NPI-Q scores were 8 and 13 respectively two and three years after baseline, including low scores in a variety of symptoms: apathy, delusions, disinhibition, irritability, sleep disturbances, loss of appetite, and aberrant motor behavior. He had to be admitted to a nursing home, experiencing further disease progression. There was full dependency in basic activities of daily living (CDR 3). He died due to a COVID-19 infection at the age of 63.

### Additional fluid biomarkers

At the time of diagnostic examination as described above, venous blood was collected, and serum aliquots were stored at the biobank of Amsterdam Dementia Cohort. [24] The patient and family gave consent to use this body material for future research purposes. Nine years later, p-tau-181*,* glial fibrillary acidic protein (GFAP), and neurofilament light (NfL) were analyzed in serum with commercially available Simoa® Assay Kits from Quanterix (analysis performed in 2022). Serum assays resulted in a low p-tau-181 concentration of 0.33 pg/ml (below the lower limit of quantification)***.*** The NfL concentration was 12.81 pg/ml and the GFAP concentration was 52.93 pg/ml. Both NfL and GFAP levels were compared to age-dependent reference ranges established by the Neurochemistry laboratory of the Amsterdam UMC. The concentration of NfL was found between the 75^th^ and 90.^th^ percentile for the control reference group, between the 10th and 25th percentile for frontotemporal dementia, and between the 25th and 50th percentile for AD. [25, 26] The level of GFAP was within the 25th and 50th percentile for the control group and below the 5th percentile for AD. [27]

### Post-mortem investigation

Before the patient’s death, relatives gave written informed consent for a post-mortem examination and pathological assessment as part of the NEurodegeneration: Traumatic brain injury as Origin of the Neuropathology (NEwTON) brain bank cohort**.** [28] Autopsy was performed within 8 h after death. Tissue was fixed in 4% formaldehyde for four weeks until macroscopic evaluation and dissection of multiple regions. Tissue blocks were embedded in paraffin and 5 μm sections were prepared for staining with Hematoxylin and Eosin (H&E), p-tau (AT8), anti-Aβ/amyloid precursor protein (APP), phosphorylated TAR DNA-binding protein (pTDP-43), alpha-synuclein, 3-repeat (3R) and 4-repeat (4R) tau (RD3, RD4), and p62. Additional staining for microglial activation (iba1 and CD68) was performed with tissue from the frontal cortex. Detailed methods of immunostaining are included in the "Additional file 1". The neuropathological evaluation was performed by an experienced neuropathologist and a pathological diagnosis was made according to international consensus guidelines [29, 30]. The preliminary NINDS/National Institute of Biomedical Imaging and Bioengineering (NIBIB) diagnostic neuropathological criteria for CTE were applied, including the recently revised staging method. [9, 12] The extent of immunoreactivity for each protein was visually assessed by two authors (SA, AJJR) and scored as none (-), minimal ( +), moderate (+ +), and extensive (+++).

### Postmortem findings

**Fig. 2:**
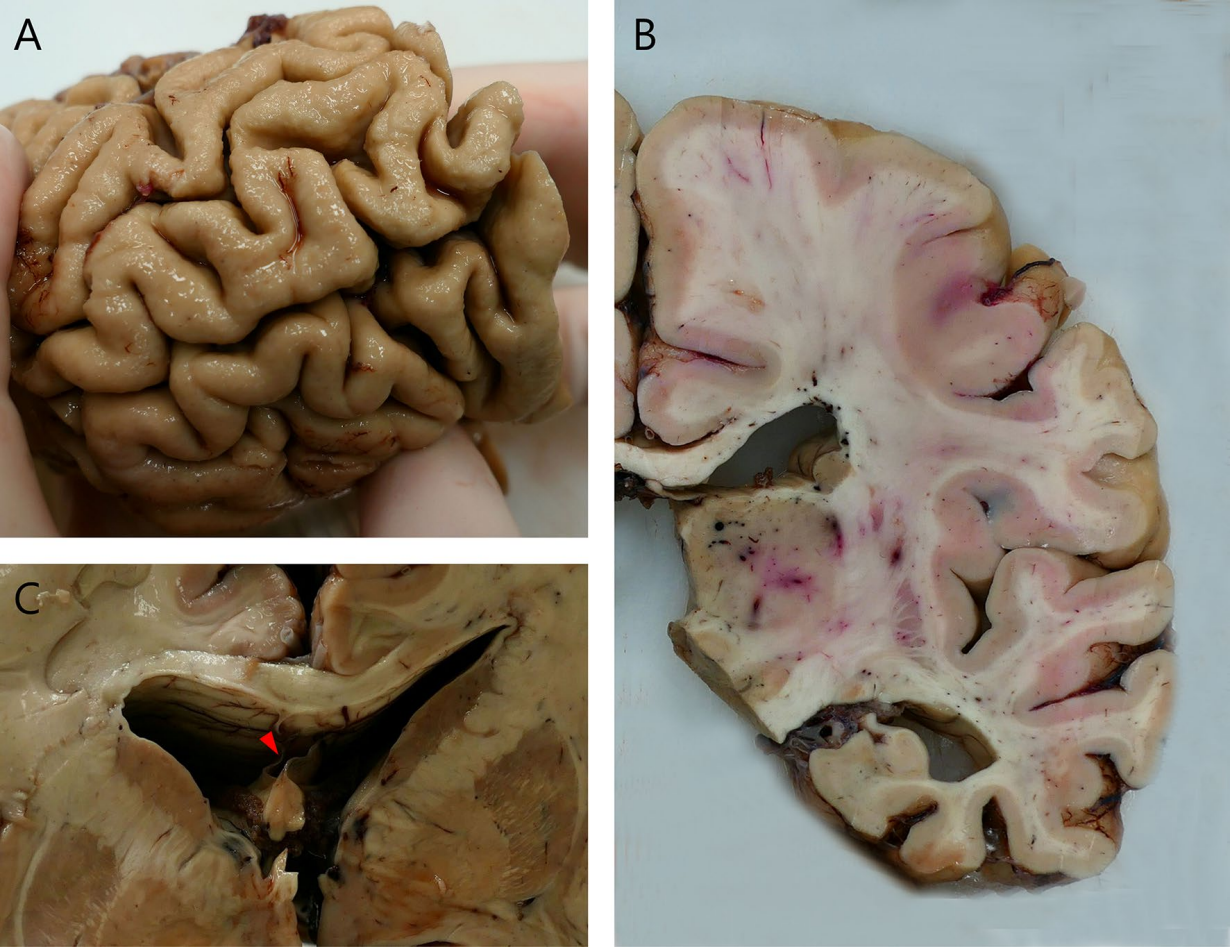
Macroscopic images. The macroscopic evaluation revealed, amongst other things, external atrophy (**a**), atrophy of the medial temporal lobe (**b**), as well as the cavum septum pellucidum (**c**, red triangle)

**Fig. 3:**
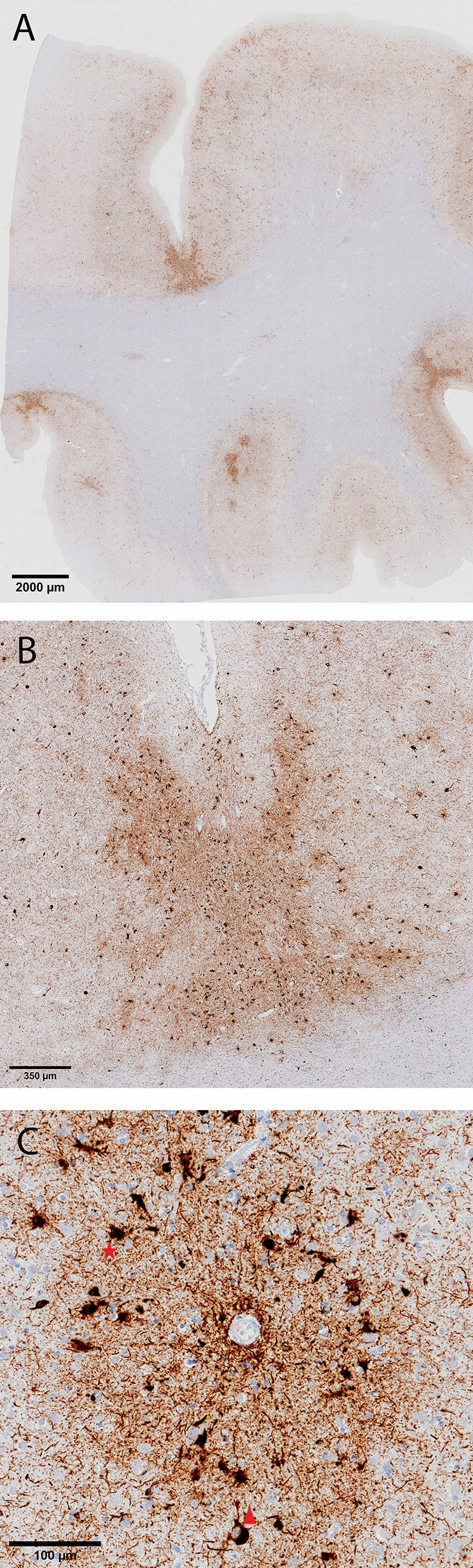
Unique CTE p-tau pathology in the frontal lobe. Overview of p-tau depositions (AT8 immunostaining) preferentially located in the sulcal depths (**a**, **b**) perivascular distribution of neuronal lesions (red triangle) and glial lesions (red asterix) (**c**)

**Fig. 4:**
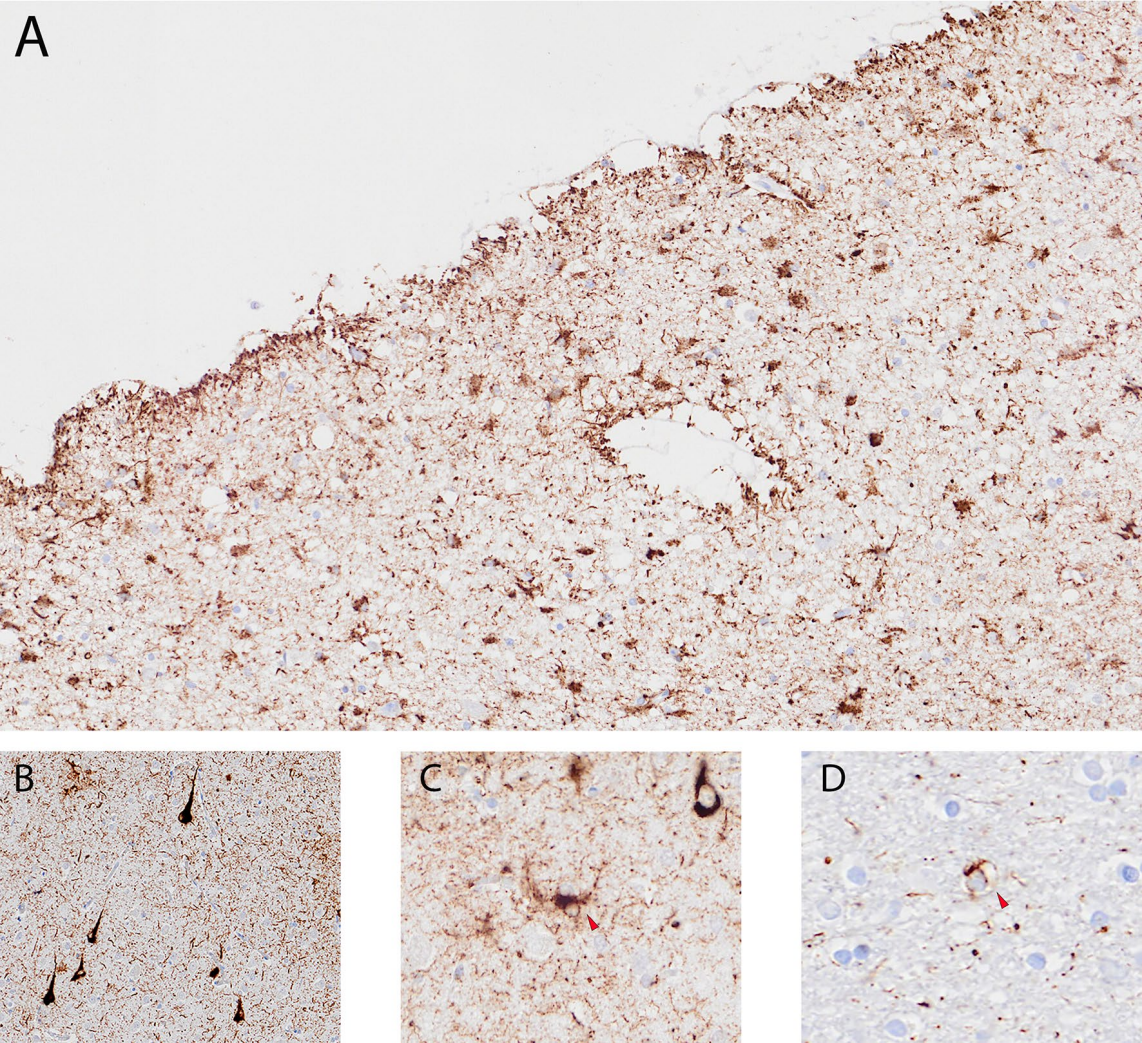
Other p-tau pathology in the frontal lobe. Thorn-shaped astrocytes immunostained for AT8, subpial and perivascular located, typical of ARTAG (**a**), neurofibrillary tangles (**b**), tufted astrocytes (**c**), coiled body in the white matter (**d**)

**Fig. 5:**
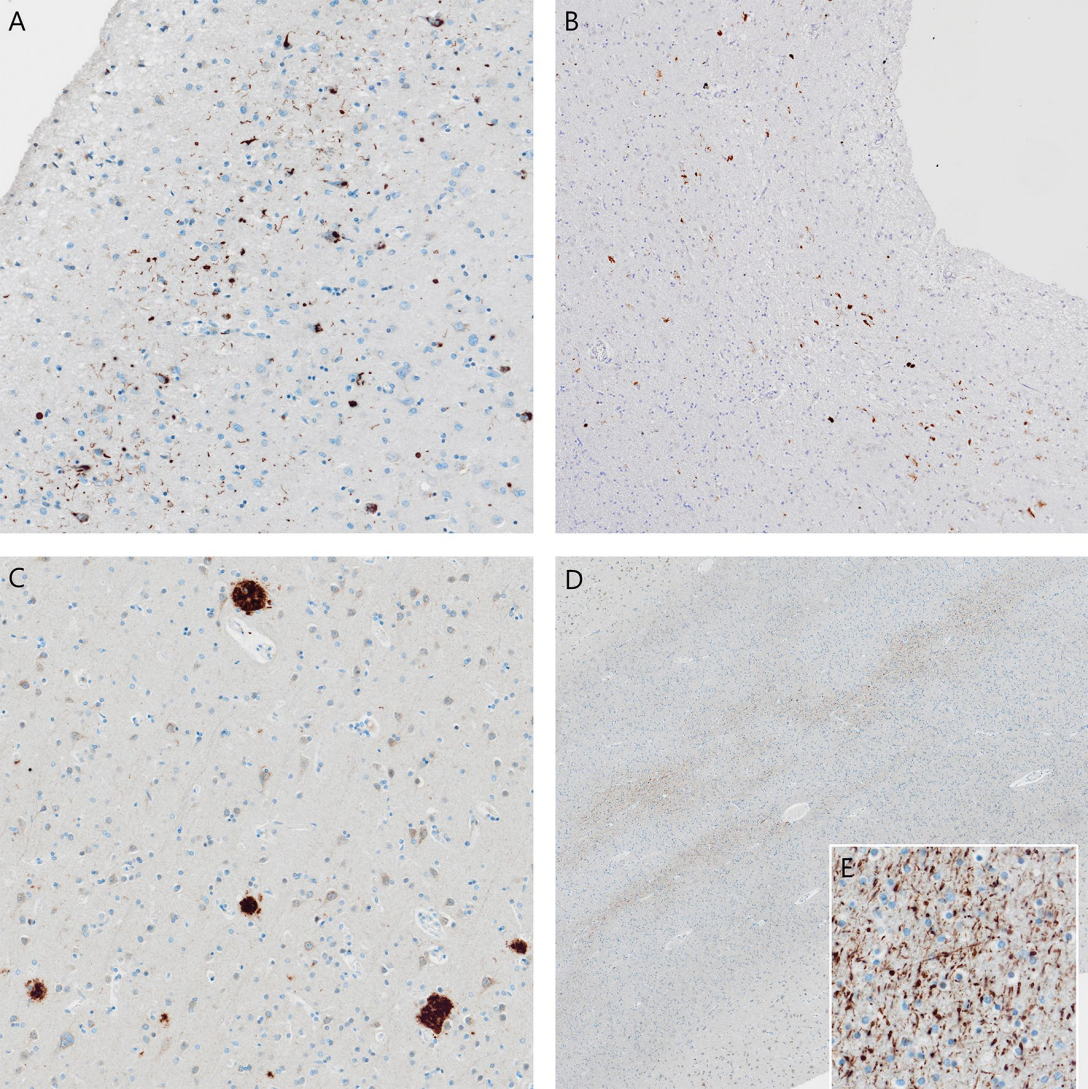
Non-tau pathology. Lesions immunostained for pTDP-43 in the hippocampus (**a**) and frontal lobe (**b**). Some diffuse plaques immunostained for Aβ in the frontal lobe (**c**). APP positivity in the white matter of the frontal lobe (**d**-**e**)

**Fig. 6:**
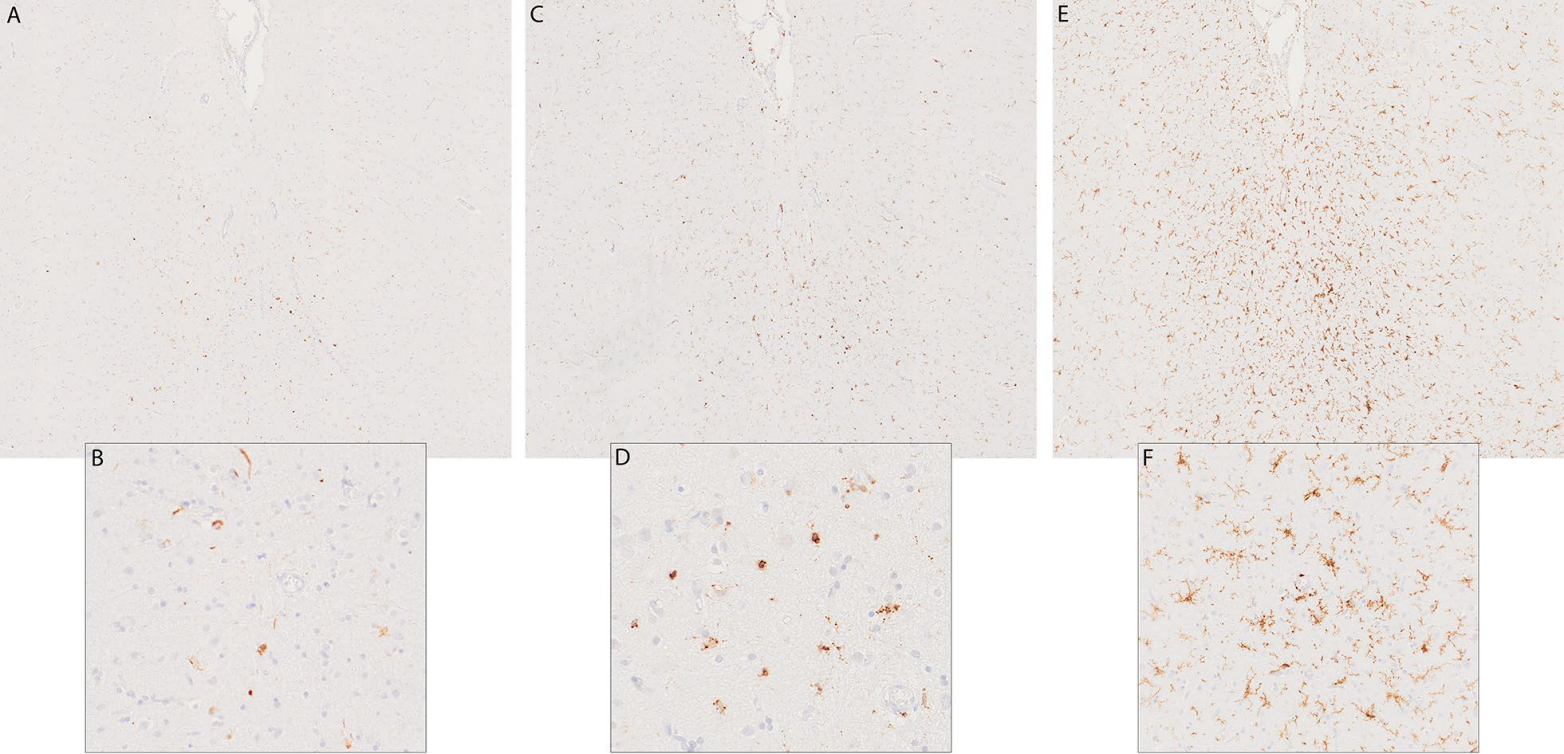
Non-tau pathology in the sulcal depths of the frontal lobe. Lesions immunostained for pTDP-43 (**a**, **b**), CD68 (**c**, **d**), Iba1 (**e**, **f**)

The fresh brain had a slightly low weight of 1220 g (excluding CSF). [31] Macroscopic examination of the whole brain (Fig. 2) showed slight external atrophy and severely widened ventricles upon dissection, especially in the frontal and temporal regions. There was atrophy of the caudate nuclei, the substantia nigra showed loss of pigment and the hippocampi appeared small. Septum abnormalities were noted as CSP and a fenestrated septum. There was only very mild atherosclerosis in the carotid arteries, without any further abnormalities in the large vessels. No macroscopic infarcts were detected. H&E staining demonstrated disturbed architecture of the second cortical layer, severe gliosis, depigmentation of the locus coeruleus, and loss of neurons in multiple cortical areas, basal ganglia, hippocampus, and dentate nucleus. The pattern of neuronal loss in the hippocampus was recognized as hippocampal sclerosis; a pathological condition that is associated with temporal lobe epilepsy but that often coexists with neurodegenerative diseases. [32] One small microscopic infarct was found in the occipital lobe, but no other evidence of cerebrovascular disease or arteriolosclerosis. P-tau staining showed widely distributed, moderate-to-extensive abnormal tau pathology throughout multiple brain regions. P-tau inclusions, including neurofibrillary tangles (NFTs), neuropil threads, thorn-shaped astrocytes, tufted astrocytes, and coiled bodies, were found in cortical and subcortical areas, as well as the cerebellum, brain stem, and cervical spine. Especially in the frontal and parietal cortex, neuronal tau pathology was found with predilection of sulcal depths and perivascular regions, according to the pathological criteria of CTE. (Fig. 3) [9] Thorn-shaped astrocytes were frequently found, located subpial, subependymal, and perivascular (Fig. 4A), as opposed to the granular/fuzzy astrocytes that were rarely present. In the hippocampus, tau positive neurons and glial cells were detected throughout all four areas (CA1-CA4). CA3 and CA4 seems to be most severely affected, but the severe neuronal loss in CA1 and CA2 complicates this assessment. Also notable were the p-tau-positive Purkinje cells in the cerebellum. According to the second NINDS/NIBIB research criteria for the diagnosis of CTE, we staged this case as high CTE, based on the additional presence of neuronal p-tau in more than 5 regions of interest (at least NFTs in gyral side and crest adjacent to CTE lesion, NFTs in CA2 and CA4 of the hippocampus, amygdala, thalamus, and cerebellar dentate nucleus). We are aware, however, of the complexity of staging in the context of mixed pathologies. [9] RD3 and RD4 staining was performed in the temporal cortex, thalamus, basal ganglia, and cerebellum, which showed both 3R and 4R tau positive neurons and coiled bodies. The glial cells in these areas were all 4R positive. There was also a moderate amount of pTDP-43 positivity in all layers of the frontal cortex, and the hippocampus including the parahippocampal gyrus with predilection of the granular layer (Fig. 5A-B). There was minimal positivity in the amygdala, the basal ganglia, and medulla. All pTDP-43 positive structures were recognized as cytoplasmic inclusions and threads, but not as neuronal intranuclear inclusions. Only a few Aβ diffuse plaques were observed in the frontal cortex but none in the hippocampus and no neuritic plaques were observed (Fig. 5C). Axonal APP positivity was found in the white matter of various regions (frontal, cerebellum, basal ganglia) (Fig. 5D-E). In addition to the AT8 positivity in the depth of the frontal cortical sulci, there was also a high density of pTDP-43, Iba1, and CD68 positive cells found in these areas, as depicted in Fig. 6. No structures immunoreactive for p62 were observed in the granular layer of the cerebellum, which makes an underlying C9orf72 hexanucleotide repeat expansion less likely. [33] In addition, no positive alpha-synuclein structures were detected in the hippocampus, amygdala, or mesencephalon. The distribution and extent of immunoreactivity for different markers are displayed in Table 1**.**

**Table 1:** Visual scores of immunoreactivity

	p-tau						3R tau			4R tau			Aβ*	APP	TDP-43	p62	α-syn
	NFT	NT	TSA	GFA	TA	CB	*neu*	*glia*	*oligo*	*neu*	*glia*	*oligo*					
Frontal	++	++	+	−	++	++							+	+++	++		
Parietal	++	++	++	+	+	+											
Temporal	+++	++	+	−	+	+	++	−	+	++	+	++					
Motor Cortex	++	+++	+	−	++	+											
Cingulate Gyrus	+	+++	+	−	+	+											
Occipital	+	+	−	+	−	−											
Hippocampus	+++	++	++	−	+	+							−	++	++		−
Amygdala	+++	++	++	+	+	–									+		−
Thalamus+STN	+	++	++	–	++	++	++	–	++	+	++	++					
Basal ganglia	+	++	+	−	++	+	++	−	++	+	+	+	−	++	+		
Hypothalamus	+	+	+	−	+	+											
Mesencephalon	+	++	+++	−	+	+											−
Substantia nigra	+	++	−	−	+	+											
Colliculus sup+inf	+	++	+++	−	++	+							+	−			
Pons+LC	++	++ +	+	−	++	++							+	−			
Medulla	+	++	+++	−	+	+									+		
Cerebellum	++	+	++	−	+	+	+	−	−	++	−	+	−	++		−	

Visual assessment of the extent of immunoreactivity for each protein, assessed by two authors. Scores: none (−), minimal (+), moderate (++), and extensive (+++)

*p-tau* phosphorylated tau, *NFT* neurofibrillary tangles, *NT* neuropil threads, *TSA* thorn-shaped astrocytes, *GFA* granular/fuzzy astrocytes; *TA* tufted astrocytes; *CB* coiled bodies, *3R* 3-repeat; *4R* 4-repeat; *neu* neuronal; *oligo* oligodendrocytes; *Aβ* amyloid-β, *APP* amyloid precursor protein, *α-syn* alpha-synuclein, *STN* subthalamic nucleus, *LC* locus coeruleus

^*^Only Aβ positive diffuse plaques were recognized, no neuritic plaques

− none, +: minimal,++: moderate,+++: extensive

## Literature review

We systematically reviewed the current literature for all confirmed CTE cases in former soccer players. We searched for literature in the PubMed and Embase databases, including the search queries: chronic traumatic encephalopathy, dementia pugilistica, neurodegeneration, soccer, (association) football, and heading (see Additional file 1). Cases were included when they fulfilled the NINDS/NIBIB preliminary neuropathological criteria for CTE and when they had a history of playing soccer. We only included papers that have been written in English. All hits (Pubmed N = 676, Embase N = 879) were independently screened for title and abstract by two authors (SA, SK). From this search, we identified 6 peer-reviewed papers and one conference abstract that involved neuropathological evaluation in soccer player(s) (total number of cases: 18). [14, 15, 34–38] We excluded four cases that lacked CTE-specific tau depositions and one case where trauma-related tau deposits were suggested but with too few NFTs to determine any preferential location. [34] This led to the identification of 13 deceased soccer players with confirmed CTE pathology. The clinical and post-mortem characteristics of these 13 individuals and the case described in this manuscript (total N = 14) are further evaluated below and summarized in Tables 2 and 3.

**Table 2:** Literature review (clinical characteristics)

Case #	Author ([14, 15, 35–38])	Year	Age at death	Age of symptom onset	Symptom duration	Sex	Position	Years of sport participation	Highest level	History of concussion	Cognitive features	Behavioral symptoms	Clinical diagnosis
1	McKee	2014	29	27	2	M	*Unknown*	19	SP	*Unknown*	*Unknown*	*Unknown*	ALS
2	Hales	2014	80	70	10	M	Forward	16^a^	P	*Unknown*	Yes	Yes	AD
3	Grinberg	2016	83	67	16	M	Defense	21^a^	P	No	Yes	Yes^b^	AD
4	Ling	2017	65	56	9	M	Forward	36	P	No	Yes	Yes	FTLD/PD
5	Ling	2017	78	69	9	M	Defense	20	P	Yes	Yes	Yes	AD/VaD
6	Ling	2017	72	63	9	M	Midfield	25	P	Yes	Yes	Yes	FTLD/AD
7	Ling	2017	83	77	6	M	Defense	20	P	Yes	Yes	Yes	AD/PD
8	Phalen	2017	24	17	7	M	*Unknown*	9	R	Yes	Yes^c^	Yes	Bipolar Disorder
9	Lee	2019	50 s	50 s	5	M	Forward	18	*Unknown*	*Unknown*	Yes	Yes	AD
10	Lee	2019	60 s	60 s	7	M	Defense	20	*Unknown*	Yes	Yes	No	AD
11	Lee	2019	70 s	60 s	8	M	Defense	35	*Unknown*	Yes	Yes	No	AD
12	Lee	2019	70 s	60 s	10	M	Defense	30	*Unknown*	No	Yes	Yes	AD/VaD
13	Lee	2019	80 s	60 s	16	M	Forward	18	*Unknown*	No	Yes	Yes^b^	AD/VaD
14	van Amerongen	2023	63	50	13	M	Forward	24	P	Yes	Yes	No	Suggestive of CTE

An overview of the clinical characteristics of soccer players with pathology-confirmed CTE

*M* male, *SP* semiprofessional, *P* professional, *R* recreational, *ALS* amyotrophic lateral sclerosis, *AD* Alzheimer’s disease, *FTLD* frontotemporal lobar degeneration, *PD* Parkinson’s disease, *VaD* vascular dementia, *CTE* chronic traumatic encephalopathy

^a^Years of participation on a professional level

^b^Behavioral symptoms only present late in the disease

^c^History did mention attention difficulties and mild memory impairment

**Table 3:** Literature review (pathological features)

Case #	Septum	CTE severity	ABC score *(*+ *ADNC)*	TDP-43	α-syn	Other pathological diagnoses
1	*Unknown*	Low	*Unknown*	Present	*Unknown*	MND
2	*Unknown*	*Unknown*	*Unknown*^a^	Present	*Unknown*	–
3	CSP	High	A1B2C3 *(intermediate)*	Present	Absent	Hippocampal sclerosis
4	CSP + F	*Unknown*	A3B2C2 *(intermediate)*	Present	Absent	CBD, CAA
5	F	*Unknown*	A2B2C2 *(intermediate)*	Present	Absent	Hippocampal sclerosis
6	F	*Unknown*	A2B2C2 *(intermediate)*	Present	Absent	CAA, Hippocampal sclerosis
7	F	*Unknown*	A3B2C2 *(intermediate)*	Present	Absent	CAA
8	CSP	*Unknown*	*Unknown*	*Unknown*	*Unknown*	*Unknown*
9	CSP + F	High	A1B2C0 *(low)*	Absent	Absent	CVD, CAA
10	CSP	High	A0B2C0 *(none)*	Present	Absent	CVD; PART
11	F	High	A2B3C3 *(intermediate)*	Absent	Present	DLB, CAA
12	*Unknown*	High	A3B3C2 *(high)*	Present	Absent	CVD; ARTAG
13	CSP	Low	A3B3C2 *(high)*	Present	Present	CVD; DLB; ARTAG; CAA
14	CSP + F	High	A1B3C0 *(low)*	Present	Absent	Hippocampal sclerosis, FTLD, CVD, ARTAG, PSP

An overview of the pathological findings of soccer players with pathology-confirmed CTE

^a^Report mentioned that amyloid plaques and neuritic plaques were present but to a lesser extent

*CTE* chronic traumatic encephalopathy, *ADNC* Alzheimer’s disease neuropathological change, *CSP* cavum septum pellucidum, *F* fenestration of septum, *MND* motor neuron disease, *CBD* corticobasal degeneration, *CAA* cerebral amyloid angiopathy, *CVD* cerebrovascular disease, *DLB* dementia with Lewy Bodies, *PART* primary age-related tauopathy, *ARTAG* aging-related tau astrogliopathy, *PSP* progressive supranuclear palsy

All cases were male and the age at death ranged between 24 and 83, with a mean symptom duration of 9.1 years (range 2–16). Most of them played soccer at a professional level for at least 16 years, as a defender (N = 6) or forward player (N = 5). Twelve out of 14 cases were clinically diagnosed with dementia and showed progressive cognitive impairment, one case presented with progressive weakness fitting a clinical diagnosis of amyotrophic lateral sclerosis and one case had a clinical diagnosis of bipolar disorder. Eight cases presented with behavioral symptoms early in their disease, two cases developed behavioral symptoms later in their disease, and only three cases described no prominent behavioral changes. Structural imaging findings (brain MRI or CT scan) were reported in three other cases, apart from the case described in this study. The CT-scan of case 2 revealed no abnormalities, case 3 demonstrated temporal atrophy and white matter hypoattenuation. The brain MRI scan of case 8 showed dilated temporal and frontal horns of the lateral ventricle and a small anterior CSP. None of the other cases had available blood and/or CSF biomarker results, or amyloid/tau PET scans. Upon post-mortem examination, 11 cases were reported to have septum pellucidum abnormalities. Severe CTE pathology was described in 6 cases, however, the severity information was missing in 6 other cases. TDP-43 proteinopathy was reported in 11 cases, including one meeting criteria for frontotemporal lobar degeneration (FTLD)-TDP and one for motor-neuron disease. Other pathology was found in terms of AD neuropathologic changes (intermediate level N = 6, high-level N = 2), cerebral amyloid angiopathy (N = 6), cardiovascular disease (N = 5), hippocampal sclerosis (N = 4), aging-related tau astrogliopathy (ARTAG) (N = 3), α-synucleinopathy/Lewy Body Disease (N = 2), corticobasal degeneration/progressive supranuclear palsy (PSP) tauopathies (N = 2), and primary age-related tauopathy (N = 1).

## Discussion

We present a comprehensive case description and post-mortem evaluation of a former Dutch professional soccer player with dementia, and we contextualize our findings through a systematic literature overview of current evidence regarding CTE and soccer. We found that, even though the clinical phenotype and the MRI scan were suggestive of AD, fluid and imaging biomarkers were unsupportive of amyloid pathology as the underlying cause of his young-onset progressive dementia. The lack of amyloid neuritic plaque pathology was confirmed at neuropathological investigation. However, the patient showed severe p-tau pathology mostly localized towards the depths of cortical sulci, fitting the NINDS/NIBIB consensus criteria of CTE [9, 12] and is suggested to be related to his extensive soccer career and exposure to RHI. A literature review revealed another 13 soccer cases with neuropathologically confirmed CTE, many with similar cognitive changes, septum abnormalities, and coexisting pathologies.

### Pathological mechanisms

Chronic traumatic changes in the brains of former athletes have been characterized for multiple decades. However, there remain many uncertainties regarding the exact pathophysiological mechanisms, the neuropathology, and its associations with RHI. Pathophysiological processes, such as diffuse axonal injury, neuroinflammation, microglial activation, and blood–brain barrier disruption all have been suggested to contribute to the onset of CTE pathology as a consequence of RHI exposure. [39] In this case, we found remarkable APP positivity, a marker for axonal injury, in the white matter. Although APP leakage has been described in brains of subjects that died after hypoxic or ischemic events, the typical pattern of hypoxic APP leakage is not observed in this case [40]. Post-mortem indications for axonal injury (including positive APP staining) have also been described in three cases with a recent COVID-19 infection, but we did not observe other acute pathologies related to COVID-19, such as hemorrhagic lesions or microvascular injury [41, 42]. APP positivity has also been reported after traumatic diffuse axonal injury, or exposure to multiple blast injuries, even years after the injury [40, 43–45]. It is possible that the APP finding in this case could be related to past RHI exposure. This needs further evaluation in future studies. We also found evidence for a close relationship between CTE pathology in the frontal sulcal depths and markers for microglial activation based on their similar preferential location (Fig. 6C-F). This is in line with previous studies, that demonstrated a direct link between elevated markers of neuroinflammation and microglial activation and p-tau pathology in brains with CTE [46, 47]. Still, we assessed the elevated protein levels only visually, and it is complicated to determine whether the activity is a consequence of RHI or neurodegeneration in general.

Our case also underscores the complexity of coexisting pathology in cases with CTE. In addition to the distinct p-tau lesions matching pathological criteria for CTE, we found multiple other tau and non-tau pathologies. The subpial, subependymal, and perivascular thorn-shaped astrocytes fit the criteria for aging-related tau astrogliopathy (ARTAG) [48] and the neuronal tau in the subthalamic nucleus and substantia nigra in combination with tufted astrocytes and coiled bodies fulfill the criteria for PSP [49–51]. Aiming to differentiate between these multiple tauopathies, we performed staining for microtubule-binding repeat domains in the temporal cortex and specific PSP regions of interest. The pattern of neuronal 3R/4R tau isomers was consistent with CTE pathology studies and not with PSP (predominant 4R tauopathy), although this could not fully exclude the (co-)presence of neuronal PSP pathology. The RD4 positive glial tau lesions were consistent with previous findings of glial tau in CTE, ARTAG as well as PSP [52–54]. We also noted cytoplasmic inclusions of TDP-43 in multiple layers of the frontal cortex, the hippocampus, basal ganglia and medulla; this distribution is consistent with the criteria of FTLD type B [55, 56]. The pattern of hippocampal and frontal TDP-43 positivity in combination with hippocampal sclerosis resembles limbic-predominant age-related TDP-43 encephalopathy (LATE), but the extensive frontal depositions and the young age makes this diagnosis unlikely. [57, 58]

Similar to other neurodegenerative diseases such as AD, mixed pathologies in CTE are common. Mez et al. [2017] reported co-pathology to be present in 45% of all CTE cases, with greater prevalence in high-stage CTE. [13] The two largest case series of CTE in soccer players revealed concomitant pathologies in all cases, recognized as AD-related changes, ARTAG, alpha-synuclein, and TDP-43 proteinopathy. [14, 15] Recently, Nicks et al. [59] demonstrated that TDP-43 inclusions (43.3%) and hippocampal sclerosis (23.4%) were prevalent in cases with CTE and that FTLD-TDP may also be present as co-pathology (6%). [59] With coexisting pathologies in neurodegenerative diseases, the question remains how to distinguish primary pathologies from secondary pathologies, and specifically in this case whether non-CTE pathologies are related to CTE p-tau pathology or whether they reflect separate processes, including those associated with RHI exposure. Nicks et al. [59] suggested that CTE may be a risk factor for the development of hippocampal sclerosis and hippocampal TDP-43, and mentioned that TDP-43 inclusions with a predilection for the sulcal depths might be a part of CTE pathology, [59] which was also noted in this case (Fig. 6 A-B). The link between RHI exposure and both ARTAG and PSP, with or without CTE p-tau pathology, has also been suggested in the past but lacks solid evidence. [60, 61] It is possible that CTE pathology accelerates the development of other pathologies, or that CTE involves a wider spectrum of pathological mechanisms and that trauma-induced processes may activate multiple pathological cascades that potentially lead to numerous neurodegenerative proteinopathies, apart from the specific p-tau lesions in the depths of the cortical sulci. Future research is necessary to disentangle these hypotheses.

## Clinicopathological correlation

The occurrence of multiple pathologies in this case complicates the clinicopathological correlation. The clinical presentation (progressive cognitive decline) resembled Alzheimer’s dementia, but this diagnosis was not supported by biomarkers and eventually excluded after neuropathological assessment. Cognitive symptoms were the most frequently reported features in RHI-exposed cases with CTE pathology, and dementia was reported in more than 50% of the CTE cases. However, formal neuropsychological test results were often unavailable, and reports mostly relied on retrospective interviews. [23, 62] Nevertheless, other major pathologies lacked strong clinicopathological correlation. The patient did not fulfil the clinical diagnostic criteria for PSP, due to the absence of ocular motor dysfunction, postural instability, and akinesia. [63] Neither did the patient meet the criteria for possible or probable behavioral variant of FTD [64], or for non-fluent variant primary progressive aphasia (the most important clinical syndromes of FTLD-TDP type B). [65, 66] ARTAG is a common pathology primarily described in the tissue of elderly brain donors and it has been suggested that ARTAG lowers the threshold for other pathologies (and related clinical impairment) to develop. [60] Still, the clinical importance of ARTAG is elusive, with studies demonstrating the lack of deficits associated with ARTAG pathology alone. [67, 68] So, it is unlikely that a single pathology fully accounted for the clinical presentation and the progressive course in this patient. Therefore, it is possible that the non-CTE pathologies (i.e., TDP-43 proteinopathy, hippocampal sclerosis), in addition to the CTE pathology, collectively contributed to the clinical disorder. Further research is needed to better understand the clinicopathological correlation of CTE and coexisting pathology in RHI-exposed individuals.

## Biomarkers

This case study once again reaffirms that research involving CTE biomarkers is still in its early stages. Amyloid PET scan results were used to evaluate the presence of neuritic amyloid plaque pathology. The negative scan was in line with previous work that demonstrated a lack of elevated amyloid plaque density in cognitively impaired American football players, [69] but had no role in demonstrating CTE or other pathology. Still, determining AD specific biomarkers in patients that have participated in contact sports may be helpful with differential diagnosis. PET scans with p-tau radiotracers may have greater potential for detecting CTE neuropathology. Flortaucipir PET scans have shown some preliminary promise, especially in late stage CTE, but there is a need for tau tracer development that is more specific to CTE tau isoforms. [69, 70] Other potential neuroimaging biomarkers are often restricted to low specificity. Like in this case, although the structural MRI scan demonstrated definite signs of neurodegeneration, the atrophy patterns were not distinguishable from other neurodegenerative diagnoses such as AD. The WMH that appeared on FLAIR MRI may be cerebrovascular-related but may also be related to RHI. Previous work demonstrated greater WMH in former football players compared to unexposed controls but the underlying etiology of these WMH needs to be unraveled in future work. [71] The CSP is also associated with RHI [72–74], frequently noted in autopsy-confirmed CTE brain donors [11], but this finding is not specific as it may also be found in the healthy population or asymptomatic contact-sport athletes. [75, 76] Consequently, the aforementioned MRI findings are potentially helpful as a marker for neurodegeneration or previous injuries rather than a specific diagnostic feature for CTE. Concerning the blood biomarkers, the low concentration of plasma p-tau-181 is consistent with recent work on two other autopsy-confirmed CTE cases that showed comparable low values of plasma p-tau-181. [77] The levels of NfL and GFAP were also relatively low, particularly in comparison with the AD population. These findings suggest that p-tau-181, and plasma NfL and GFAP may have less potential for assessing tau and non-tau-related pathologies in CTE and that other tau epitopes may have greater potential. This needs further evaluation in a larger sample of pathology confirmed CTE cases.

## Conclusions

The relationship between soccer and neurodegeneration is increasingly recognized in clinical studies, but less supported by pathological studies with very few post-mortem confirmed CTE cases in the literature. This study adds to the current literature and will hopefully increase awareness and appreciation of the complexity of the clinicopathological correlation and diagnosis of CTE during life. Large-scale clinicopathological research among former soccer players is necessary for a better understanding and to find out the exact prevalence and risk factors for CTE in this population.

## Supplementary Information

**Additional file 1. Supplementary Materials.** This file includes a detailed description of the immunostaining methods and the search queries for the literature search.

## Data Availability

Data sharing does not apply to this article as no datasets were generated or analyzed during the current study. Material may be available upon reasonable request.

## Abbreviations

3R: 3-Repeat
4R: 4-Repeat
AD: Alzheimer's disease
ALS: Amyotrophic lateral sclerosis
APP: Amyloid precursor protein
ARTAG: Aging-related tau astrogliopathy
Aβ: Amyloid-beta
α-syn: Alpha-synuclein
C9orf72: Chromosome 9 open reading frame 72
CAA: Cerebral amyloid angiopathy
CB: Coiled bodies
CBD: Corticobasal degeneration
CDR: Clinical Dementia Rating
CSF: Cerebrospinal fluid
CSP: Cavum septum pellucidum
CTE: Chronic traumatic encephalopathy
CVD: Cardiovascular disease
DAB: 3,3′-Diaminobenzidine tetrahydrochloride
DLB: Dementia with Lewy Bodies
FLAIR: Fluid-attenuated inversion recovery
FTLD: Frontotemporal lobar degeneration
GFAP: Glial fibrillary acidic protein
GFA: Granular/fuzzy astrocytes
H&E: Hematoxylin and Eosin
IHC: Immunohistochemistry
LATE: Limbic-predominant age-related TDP-43 encephalopathy
LC: Locus coeruleus
MAPT: Microtubule-associated protein tau
MMSE: Mini-mental state examination
MND: Motor neuron disease
MRI: Magnetic resonance imaging
NBB: Netherlands brain bank
neu: Neuronal
NEwTON: Neurodegeneration: Traumatic brain injury as Origin of the Neuropathology
NfL: Neurofilament light
NFTs: Neurofibrillary tangles
NIBIB: National Institute of Biomedical Imaging and Bioengineering
NINDS: National Institute on Neurological Disorders and Stroke
NPI-Q: Neuropsychiatry Inventory Questionnaire
NT: Neuropil threads
oligo: Oligodendrocytes
PART: Primary age-related tauopathy
PD: Parkinson's disease
PET: Positron emission tomography
PSP: Progressive supranuclear palsy
p-tau: Phosphorylated tau
RHI: Repetitive head impacts
STN: Subthalamic nucleus
TA: Tufted astrocytes
(p)TDP(-43): (Phosphorylated) TAR DNA-binding protein (43)
TES: Traumatic Encephalopathy Syndrome
TSA: Thorn-shaped astrocytes
UMC: University Medical Centers
VaD: Vascular dementia
VU: Vrije Universiteit
WMH: White matter hyperintensities
α-syn: Alpha-synuclein
