# Supplementary material for: Severe CTE and TDP-43 pathology in a former professional soccer player with dementia: a clinicopathological case report and review of the literature
Source: Acta Neuropathol Commun. 2023 May 10;11:77. doi: 10.1186/s40478-023-01572-3 (PMC10169296; doi:10.1186/s40478-023-01572-3)
Supplement: Supplementary file 1 — Additional file 1. Supplementary Materials. This file includes a detailed description of the immunostaining methods and the search queries for the literature search. [file 40478_2023_1572_MOESM1_ESM.docx]

**Supplementary materials**

**Immunostaining methods**

*Immunohistochemistry (IHC)*

IHC was performed using the Ventana BenchMark ULTRA staining system (Roche, Basel, Switzerland). Tissue sections were mounted on TOMO adhesive glass slides (Matsunami, Osaka, Japan) and deparaffinized. After blocking for endogenous peroxidase, antigen retrieval was performed by heating sections at 100°C in Cell Conditioning 1 solution (pH 8.5) (Roche). For detection of primary antibodies (see Suppl. Table 1) with 3,3’-diaminobenzidine tetrahydrochloride (DAB), Optiview DAB IHC detection kit (Roche) was used. Finally, sections were mounted with coverslipping film (Sakura Tissue-Tek, Leiden, The Netherlands).

Suppl. Table 1. Characteristics of primary antibodies

| Antibody | Antigen | Species | Dilution | Origin details |
| --- | --- | --- | --- | --- |
| p-tau | Tau phosphorylated at Ser202 and Thr205, clone AT8 | Mouse IgG1 | 1:10.000 | ThermoFisher, Pittsburgh, USA |
| Aβ | N-terminus of amyloid-beta (aa 1-16), clone IC-16 | Mouse IgG2a | 1:25 | Dr. Carsten Korth, University of Dusseldorf |
| pTDP-43 | Anti TAR DNA-Binding Protein 43 (TDP43), phospho Ser409/410 | Mouse | 1:8000 | Cosmo Bio Co, Tokyo, Japan |
| α-syn | Lewy bodies, clone LB509 | Mouse | 1:200 | Invitrogen, Thermo Fisher Scien- tific, MA, USA |
| 3R tau | 3-repeat region, 3-repeat tau isoform, clone 8E6/C11 | Mouse IgG | 1:12.000 | Merck-Millipore, MA, USA |
| 4R tau | 4-repeat region, 4-repeat tau isoform, clone 1E1/A6 | Mouse IgG | 1:200 | Merck-Millipore, MA, USA |
| P62 | Human p62 lck ligand aa. 257-437 | Mouse IgG1k | 1:1000 | BD Transduction Laboratories, Allschwil, Switzerland |
| Iba1 | C-terminus of Iba1 | Rabbit | 1:4000 | Wako Pure Chemical Industries, Osaka, Japan |
| CD68 | CD68, clone KP1 | Mouse IgG1 | 1:1200 | Dako, Glostrup, Denmark |

**Literature search**

Search terms (last update September 26, 2022)

(CTE OR "chronic traumatic encephalopathy" OR dementia OR pugilistica OR "punch drunk" OR neurodegenerati*) AND (soccer OR football OR "association football" OR heading)
